# Sinus histiocytosis with massive lymphadenopathy (Rosai-Dorfman Disease): A case report and review of 49 cases with fine needle aspiration cytology

**DOI:** 10.4103/1742-6413.76731

**Published:** 2011-02-12

**Authors:** Yuquan Shi, Adrienne Carruth Griffin, Paul JL Zhang, James N Palmer, Prabodh Gupta

**Affiliations:** 1 Department of Pathology and Laboratory Medicine, Pennsylvania Hospital, Philadelphia, PA, USA; 2 Hospital of the University of Pennsylvania, Philadelphia, PA, USA; 3 Department of Otorhinolaryngology, Head and Neck Surgery Rhinology Division, Hospital of the University of Pennsylvania, Philadelphia, PA, USA

**Keywords:** Fine needle aspiration, Rosai-Dorfman disease, Sinus histiocytosis with massive lymphadenopathy

## Abstract

Rosai–Dorfman disease (RDD), a rare, benign, self-limiting histiocytic proliferative disorder, can be encountered in both nodal and extranodal locations, and fine needle aspiration (FNA), a simple, accurate and economic tool, has been widely used for the diagnosis of superficial and deep-seated lesions. Familiarity with the cytomorphologic features of RDD is important as prognosis and treatment are quite different from other benign or malignant diseases for which it may clinically masquerade. Although large numbers of RDD cases have been reported, review of the literature has revealed 49 reported cases of RDD diagnosed by FNA. Here, we report a case of RDD with nasal and sinus involvement. The patient was seen at our institution, carrying a diagnosis of inflammatory pseudotumor rendered by an outside institution, based on material obtained by nasal and sinus surgical biopsies. Cervical lymph node FNA performed at our institution revealed typical features of RDD. The case, as well as a brief review of the literature and 49 RDD cases with FNA cytology, will be discussed.

## CASE REPORT

**Figure 1:**
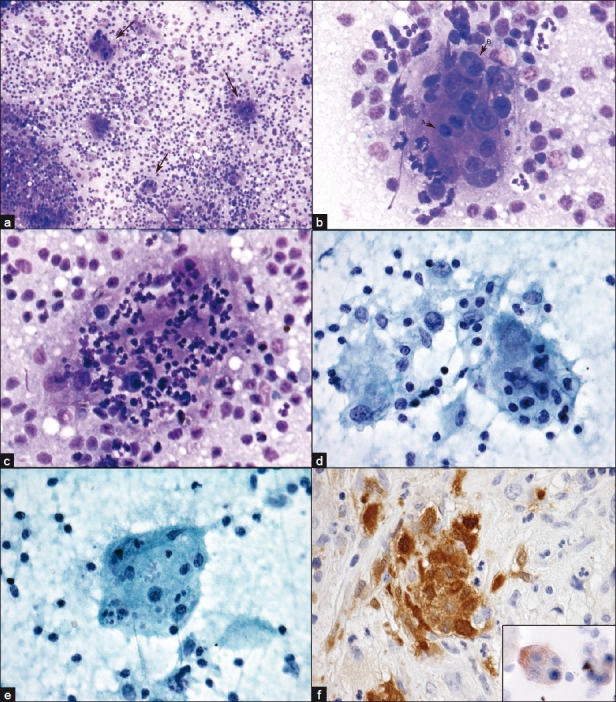
Emperipolesis, RDD. FNA cervical lymph node. Emperipolesis in multinucleated giant cells (arrows) LP (a). Lymphocytes (arrow A) and plasma cells (arrow B) HP (b). Neutrophils and a few plasma cells (c) HP. Lymphocytes, neutophils, plasma cells and associated macrophages (d), HP. Lymphocytes and nuclear debris (e) high power. Nasal biopsy with S-100 positive cells, insert cytospin specimen with S-100 positive multinucleated giant cells. a–c) Diff Quik®; d-e) Papanicolaou stain; f) immunoperoxidase stain with DAB

The patient was a 79-year-old female who presented at an outside hospital with 2 years of nasal obstruction and epistaxis. She underwent surgery for removal of a right middle turbinate mass. Biopsies of a right septal lesion and the right ethmoid sinus were also performed at the same time. Pathologic findings noted were “spindle cell proliferation with abundant foamy macrophages and inflammatory cells including plasma cells and neutrophils.” Special stains for acid-fast bacilli, fungi and bacteria were all negative. An immunohistochemical stain for cytokeratin showed no immunoreactivity in the spindle cell component. Flow cytometry showed no evidence of B-cell or T-cell lymphoma. A diagnosis of “inflammatory pseudotumor (inflammatory myofibroblastic tumor)” was suggested by the outside institution. After surgery, the patient continued to experience nasal obstruction as well as alternating bilateral epistaxis and she eventually sought a second opinion at the Hospital of the University of Pennsylvania. Her physical examination was notable for bilaterally enlarged (3–5 cm), soft, smooth, mobile and non-tender submandibular lymph nodes. A fine needle aspiration (FNA) was performed on a left submandibular lymph node, and an onsite Diff Quik stained direct smear revealed numerous multinucleated giant cells with extensive emperipolesis in the background of mixed inflammatory cells [Figure 1]. An onsite, preliminary interpretation was reported as “submandibular lymph node aspiration with features suggestive of Rosai-Dorfman disease (RDD).” Subsequent specimen processing with Papanicolaou stained smears and a detailed examination of the Diff Quik smears revealed numerous multinucleated giant cells in a lymphohistiocytic background. Lymphocytes included larger and immature forms along with numerous neutrophils, a few plasma cells and rare eosinophils. The multinucleated giant cells showed anisocytosis, up to 80–100 μm in size, with vesicular, small monotonous nuclei. Emperipolesis of numerous neutrophils, variable numbers of lymphocytes and occasional plasma cells was noted. Ingested cells were well preserved; no mitosis or necrosis was observed. Immunocytochemical staining with appropriate controls was performed on cytospin preparations and revealed the multinucleated giant cells as positive for S100 and negative for CD1a. A diagnosis of sinus histiocytosis with massive lymphadenopathy (SHML; also known as RDD) was made. Review of the outside slides from the nasopharyngeal region revealed a mixed inflammatory infiltrate extensively involving the sinonasal mucosa and squamous lined mucosa with a moderate increase in fibrous tissue. Notably, scattered histiocytes with slightly atypical nuclei and ample foamy cytoplasm contain trafficking lymphocytes and neutrophils (emperipolesis), though not as prominently identified as in the lymph node FNA specimen. Additional immunohistochemical staining performed on the surgical biopsy material demonstrated that the histiocytes were positive for S100 and negative for CD1a, CD30 and ALK. A diagnosis of RDD involving the right middle turbinate, right nasal septum and right ethmoid sinus was subsequently rendered. Presently, the patient is being treated with prednisone. Additional sinus biopsies were performed at the Hospital of the University of Pennsylvania, which demonstrated the same morphologic and immunohistologic features as described above, lending further support for the diagnosis of extranodal RDD.

## DISCUSSION

Sinus histiocytosis with massive lymphadenopathy (SHML), also known as RDD, is a rare nonmalignant proliferative disorder first described by Rosai and Dorfman in 1969. Classically, it presents as prominent bilateral, massive, painless cervical lymphadenopathy; low-grade fever, weight loss, leukocytosis, elevated erythrocyte sedimentation rate and hypergammaglobulinemia are often found. Extranodal involvement of at least one site is identified in 43% of RDD cases and only 23% exclusively have extranodal disease.[1] Documented sites of extranodal involvement include skin, respiratory tract, bone, genitourinary system, oral cavity, central nervous system, eyes/orbit/ocular adnexa, salivary gland, tonsil, breast, soft tissue and heart. The bone marrow is rarely involved.[2] Most patients with RDD have a complete and spontaneous remission. Some may experience recurrent or persistent but stable lymphadenopathy. In rare cases, the disease follows an aggressive course and may be fatal[3] and involvement of kidney, lower respiratory tract, or liver has been found to be a poor prognostic sign.[1] Occasionally, RDD may be associated with autoimmune disorders and hematopoietic malignancies.[4–7] The etiology of RDD is unknown though it has been speculated that an occult chronic infection or an aberrant exaggerated immune response to an infectious agent or an antigen causes the initial histiocytic proliferation.[3]

RDD is notable for its varied clinical presentations which evoke a wide differential diagnosis. Although correlation of clinical presentation with radiologic and laboratory values is very helpful, the pathologic assessment is pivotal in making the diagnosis. The classic histology is characterized by effacement of nodal architecture and dilatation of lymph node sinuses by lymphocytes, plasma cells and numerous characteristic histiocytes with large vesicular nuclei and abundant clear cytoplasm. Many of these histiocytes, also known as RDD cells, contain intact lymphocytes, and sometimes plasma cells and red blood cells, within their cytoplasm. This process whereby cells enter and transit through a cell evading cellular degradation is known as emperipolesis and was first described by Humble *et al*.[8] When extranodal sites are involved, similar morphologic features to the nodal counterpart are seen although with more fibrosis, fewer typical RDD histiocytes, and less prominent emperipolesis.[3] Immunohistochemical stains are useful when diagnosing RDD as the RDD cells have been found to express pan-macrophage antigens (CD68, HAM56, CD14, etc.), antigens associated with phagocytosis (CD64, Fc receptor for immunoglobulin G), lysosomal activity (lysozyme alpha 1-antitrypsin, alpha1-antichymotrypsin), and immune activation and adhesion molecules (transferring receptor, interleukin 2 receptor).[3] The most consistent and reliable phenotype for RDD is S100(+), CD68(+) and CD1a(−).

RDD can be encountered in both nodal and extranodal locations, and FNA, a simple, accurate and economic tool, has been widely used for the diagnosis of superficial and deep-seated lesions. Familiarity with the cytomorphologic features of RDD is important as prognosis and treatment are quite different from other benign or malignant diseases for which it may clinically masquerade. Review of the literature has revealed 49 reported cases of RDD diagnosed by FNA [Table 1]. Typically, FNA specimens show non-cohesive, diffusely distributed, enlarged histiocytes. These cells have variable nuclei, abundant cyanophilic cytoplasm and demonstrate emperipolesis of lymphocytes, plasma cells and occasionally erythrocytes.[19,33] Although the nuclear shapes vary from round to extremely bizarre configurations, the chromatin is fine and evenly distributed and the nucleoli are usually not prominent. Occasionally, atypical morphology may be seen and, when present, it can lead to a misdiagnosis of malignancy.[19] Lymphocytes, plasma cells, neutrophils and follicular center cells are often found in the background of the smear.[19,33]

**Table 1:** Rosai–Dorfman disease: Summary of 49 cases with fine needle aspiration

*Case*	*Author*	*Publication year*	*Sex (M/F)*	*Age (years)*	*FNA site*	*FNA diagnosis*	*Surgical diagnosis*	*Immunostains*
1	Pettinato[9]	1990	(1/0)	3	LN	RDD	No	S100+, AIAT+, LYZ− (FNA)
2	Layfield[10]	1990	(1/0)	5	LN	Reactive lymphoid infiltrate with benign histiocytes	RDD	No
3	Trautman[11]	1991	(1/0)	14	LN	RDD	RDD (larynx)	No
4	Schmitt[12]	1992	(1/0)	12	LN	RDD	RDD	S100+, HAM56+, LYZ−, AlAT+ (biopsy)
5	Schmitt[12]	1992	(1/0)	15	LN	RDD	RDD	S100+, HAM56+, LYZ+, AlAT+ (biopsy)
6	Schmitt[12]	1992	(1/0)	18	LN	RDD	RDD	S100+, HAM56+, LYZ−, AlAT (biops
7	Chang[13]	1993	(1/0)	30	LN	RDD	RDD	No
8	Perez-Guillermo[14]	1993	(0/1)	71	Breast	RDD	RDD	No
9	Alvarez[15]	1995	(0/1)	62	LN	RDD	RDD	S100+, LYZ+ (biopsy)
10	Gupta[16]	1996	(1/0)	12	LN	RDD	RDD	No
11	Patel[17]	1996	(1/0)	12	LN	RDD	RDD	No
12	Norman[18]	1997	(1/0)	71	Parotid	Suggestive of malignancy	RDD	No
13	Deshpanande[19]	1998	(1/0)	40	LN	RDD	No	No
14-19	Deshpanande[19]	1998	(4/2)	1.5–40	LN	RDD	RDD	No
20	Deshpanande[19]	1998	(1/0)	21	LN	Suggestive of Hodgkin’s lymphoma	RDD	No
21	Hummel[20]	1999	(0/1)	52	Breast	Atypical lymphohistiocytic proliferation	RDD	No
22	Soares[21]	1999	(0/1)	65	Breast	Centroblastic non-Hodgkin’s malignant lymphoma	RDD	No
23-26	Deshpande[22]	2000	(3/1)	12–17	LN	RDD	RDD	No
27	Juskevicius[23]	2001	(0/1)	48	Parotid	RDD	RDD	S100+, HAM56+, CDla- (biopsy)
28	Das[24]	2001	(0/1)	14	LN	RDD	No	No
29	Das[24]	2001	(0/1)	35	LN	RDD	No	S100+ (FNA)
30	Goel[25]	2003	(0/1)	7	Bone	RDD	No	No
31	Cocker[26]	2003	(0/1)	38	Thyroid	Lymph node	RDD	S100+ (biopsy)
32	Singh[27]	2004	(0/1)	35	Skin	RDD	RDD	S100+, CD68+, CDIIc+, CDIa- (FNA)
33	Panikar[28]	2005	(0/1)	45	LN	RDD	No	No
34	Ruggiero[29]	2006	(0/1)	12	LN	RDD	No	S100+, CD68+
35	Ruggiero[29]	2006	(1/0)	9	LN	RDD	No	S100+, CD68+, CDIa- (FNA)
36	Maiur[30]	2007	(0/1)	50	Orbit	RDD	RDD	No
37	Sachdev[31]	2007	(0/1)	47	LN	RDD	No	S100+, CD68+, CDIa- (FNA)
38	Bist[32]	2007	(1/0)	20	LN	RDD	RDD (nasal mass)	No
39, 40	Kumar[33]	2008	(0/2)	35–50	LN	RDD	RDD	No
41, 42	Kumar[33]	2008	(2 0)	6–75	LN	RDD	No	No
43	Pinto[34]	2008	(1/0)	43	LN	Non-conciusive	RDD	No
44	Jing[35]	2009	(0/1)	51	Bone	Acute and chronic inflammation	RDD	S100+, CDIa-(biopsy)
45	Farkash[36]	2009	(0/1)	52	LN	RDD	RDD	No
46	Tseng[37]	2009	(1/0)	55	Subglottis	RDD	RDD	No
47	Morkowski[38]	2010	(0/1)	53	Breast	Reactive lymphoid hyperplasia	RDD	S100+, CD68+, CDIa-(excision)
48	Bansai[39]	2010	(1/0)	35	Breast	RDD	No	No
49	Li[40]	2010	(0/1)	28	Bone	RDD	No	S100+, CDIa- (FNA)

LN, Lymph node; FNA, Fine needle aspiration; RDD, Rosai-Dorfman Disease

**Figure 2:**
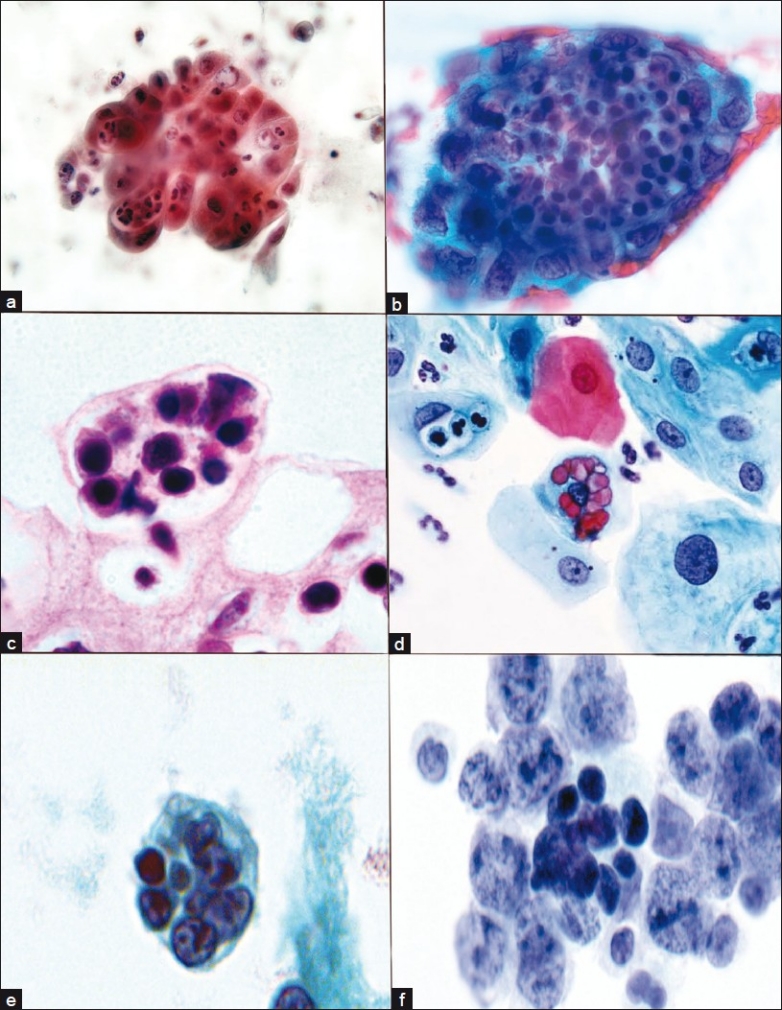
Emperipolesis in other disorders. Acute and chronic cervicitis. Endocervical cells with indigested neutrophils, cervical smear (a). Endometrial adenocarcinoma tissue fragment with intracytoplasmic lymphocytes and neutophils, cervicovaginal smear (b). Intracytoplasmic lymphocytes, cytomegalovirus (CMV) infection, cervical biopsy (c). Ingested erythrocytes metaplastic endocervical cells, HSV, cervical smear (d). Ingested lymphocytes and debris, lymphoma cerebrospinal fluid (e). Ingested lymphocytes, B cell lymphoma, cervical lymph node FNA (f). (a, b, d, e, f) Papanicolaou; (c) H/E stain

Misdiagnosis of RDD can be rendered in both FNA and surgical biopsy specimens. When compared to surgical core or excisional biopsy, FNA can at times be misinterpreted due to limited or non-representative sampling and, as FNA does not permit examination of the tissue architecture, diagnosis can be further confounded. Despite these potential limitations, FNA is still a very useful tool for the diagnosis of RDD. In fact, emperipolesis, the hallmark for RDD, tends to be more prominent in FNA material than on tissue sections. This is believed to be due, in part, to the fact that in FNA smears, an entire cell may be observed by focusing through the different planes of the slide, whereas in surgical pathology sections, generally a single plane of tissue is available for review. Furthermore, the physical action of spreading the sample into a smear of single cells renders the cellular borders of histiocytes more clearly defined and, thereby, emperipolesis may be more readily and definitively identified. It is important to note, however, that the occurrence of emperipolesis per se should not be considered diagnostic of RDD. Instead, diagnosis requires correlation of the appropriate clinical presentation with an adequately preserved and properly prepared FNA sample of consistent cytomorphologic features. In addition, numerous other disease states either demonstrate emperipolesis or represent cytomorphologic mimics, including lymphoma, malignancy, hemophagocytic syndrome, infection, Langerhans histiocytosis and various other reactive processes [Figure 2].

Significantly, misdiagnosis of RDD by FNA more often occurs in extranodal rather than in nodal disease. In the cases with biopsy confirmation [Table 1], 3 out of 25 (12%) lymph node aspirations were misdiagnosed or inconclusive and 6 out of 12 (50%) extranodal aspirations were misdiagnosed. Therefore, in the case where extranodal RDD is suspected, careful clinical lymph node examination should be undertaken and FNA performed in order to maximize diagnostic accuracy. Finally, as RDD is infrequently suspected clinically and is a rare disease process, an awareness of the entity along with its clinical profile is essential in proper specimen evaluation, interpretation and diagnosis.

## CONCLUSION AND SUMMARY

In summary, RDD is a rare disease of unknown etiology, typically presenting in young adulthood and following a relatively benign clinical course. As a diagnostic entity it is significant in that it has the readily recognizable, although not specific, cytomorphologic feature of emperipolesis on FNA. This finding should also bring to mind a wide differential diagnosis including lymphoma, malignancy, hemophagocytic syndrome, infection, Langerhans histiocytosis and various other reactive processes. Lymph node specimens are most helpful diagnostically and should be sampled, in addition to extranodal sites, when possible. FNA represents an efficient, minimally invasive, cost-effective and reliable, though not infallible, technique for the diagnosis of RDD. However, awareness of the entity and consideration of it as a diagnosis in the review and histiocytic and lymphocytic pathologies is of utmost importance if one is to be successful in the diagnosis of RDD by FNA.

## COMPETING INTEREST STATEMENT BY ALL AUTHORS

No competing interest to declare by any of the authors.

## AUTHORSHIP STATEMENT BY ALL AUTHORS

Each author acknowledges that this final version was read and approved. All authors qualify for authorship as defined by ICMJE http://www.icmje.org/#author. Each author participated sufficiently in the work and takes public responsibility for appropriate portions of the content of this article.

## ETHICS STATEMENT BY ALL AUTHORS

This study was conducted with approval from institutional Review Board (IRB) (or its equivalent) of all the institutions associated with this study.

## EDITORIAL / PEER-REVIEW STATEMENT

To ensure integrity and highest quality of CytoJournal publications, the review process of this manuscript was conducted under a double blind model(authors are blinded for reviewers and reviewers are blinded for authors)through automatic online system.
